# Is videoconference pulmonary rehabilitation associated with improvements in knowledge in people living with COPD? A propensity-matched service-evaluation

**DOI:** 10.1177/14799731241310895

**Published:** 2025-01-22

**Authors:** Ching Yee Cheung, Wing Shing Yam, Melanie D Palmer, Stuart Clarke, William DC Man, Nicola J Roberts, Claire M Nolan

**Affiliations:** 1 3890 Brunel University London, College of Health Medicine and Life Sciences, London, UK; 2 Harefield Pulmonary Rehabilitation Unit, Harefield Hospital, 8945 Guy’s and St Thomas’ NHS Foundation Trust, London, UK; 3 Department of Respiratory Medicine, Harefield Hospital, 8945 Guy’s and St Thomas’ NHS Foundation Trust, London, UK; 4 National Heart and Lung Institute, Imperial College London, London, UK; 5 King’s Centre for Lung Health, Faculty of Life Sciences and Medicine, Kings College London, London, UK; 6 School of Health and Social Care, 3121 Edinburgh Napier University, Edinburgh, UK

**Keywords:** COPD, Videoconference pulmonary rehabilitation, pulmonary rehabilitation, knowledge, lung information needs questionnaire

## Abstract

**Introduction:** Pulmonary rehabilitation (PR) services are increasingly using alternative programme delivery modes, for example telerehabilitation strategies including videoconferencing, to improve patient choice and accessibility. Although telerehabilitation results in improvements in core outcomes, the effect on knowledge attainment is not known. **Aim:** To observe the real-world responses of patients choosing to undergo videoconference PR to a matched control group choosing to undergo in-person PR, in terms of knowledge attainment. **Methods:** Using propensity score matching, 25 people with COPD who completed videoconference PR were matched 1:1 with a control group of 25 people with COPD who completed in-person PR. Knowledge attainment was measured using the Lung Information Needs Questionnaire (LINQ). **Results:** There was a statistically and clinically significant improvement in LINQ score in both groups (mean (95%CI): videoconference −3.2 (−4.7 to −1.6); in-person −3.0 (−4.5 to −1.4)), with no significant between-group difference (mean (95%CI): 0.2 (−2.0 to −2.4)). 76% and 80% of participants achieved the minimal important difference of the LINQ in the videoconference and in-person PR groups respectively. **Conclusion:** In conclusion, this real-world service evaluation indicates that videoconference PR may be associated with similar improvements in knowledge attainment as in-person PR, but this requires corroboration due to the small sample size.

## Introduction

Pulmonary rehabilitation (PR) is a core COPD management strategy that uses exercise and education to improve physical and psychological function, and promote effective self-management.^1^ National and international guidelines recommend the inclusion of structured education sessions in PR programmes as knowledge attainment is fundamental to facilitating positive behavioural changes and self-management.^1^

There is growing interest in alternative PR delivery modes, e.g. telerehabilitation strategies including videoconference, to increase patient choice and programme accessibility. Although PR delivered using telerehabilitation results in similar improvements to in-person PR for exercise capacity, breathlessness and health-related quality of life, the effect on knowledge attainment has not been investigated.^2^ Therefore, the aim of this service evaluation was to observe the real-world responses of people with COPD choosing to undergo videoconference PR to a matched control group choosing to undergo in-person PR, in terms of knowledge attainment. We hypothesised that there would be similar improvements in knowledge attainment in both PR groups because despite a different delivery format, the same education material would be delivered.

## Methods

Participants were people living with COPD who had completed PR at Harefield Hospital PR Unit, UK between 2020 and 2022. In line with clinical practice, individuals with comorbidities that would make exercise unsafe were excluded from participating in PR (e.g. unstable angina). All participants provided informed consent to participate in PR and for their anonymised data to be used for service evaluation purposes. The evaluation was conducted in line with the Declaration of Helsinki.

All participants underwent an in-person pre-PR assessment and selected videoconference or in-person PR. To balance participant baseline characteristics, 1:1 propensity score matching, using the nearest neighbour method, was used to account for age, sex and Lung Information Needs Questionnaire (LINQ).

Videoconference and in-person PR involved 8 weeks of twice weekly supervised sessions. Both programmes involved 1 hour of group-based individually tailored aerobic and resistance exercise supervised by a physiotherapist and physiotherapy assistant. The education component of both programmes involved 30 minutes of group-based structured education using a combination of lectures (*n* = 11) delivered by the physiotherapist and pre-recorded videos (*n* = 5) delivered by members of the multidisciplinary team, supplemented by quizzes, interactive scenarios and question-and-answer sessions. The physiotherapist led the question-and-answer sessions after playing the pre-recorded videos delivered by the multidisciplinary team and if they didn’t know the answer to a question, they asked the relevant professional after the session and told the group the answer at the subsequent session. The education content based on national guidelines and supplemented with an education booklet.

Outcomes were measured at in-person assessments pre- and post-PR. The primary outcome was the LINQ, a self-completed 16-item questionnaire that measures knowledge and education needs.^3^ Additional outcomes included measures of breathlessness (Medical Research Council dyspnoea scale: MRC), health-related quality of life (Chronic Respiratory Questionnaire: CRQ) and exercise capacity (incremental shuttle walk test: ISW).

Data analysis included descriptive analyses and Paired Samples *t* test (or Wilcoxon Signed-Rank test), Independent Samples *t* test (or Mann–Whitney U test) or Chi-Square test to evaluate within- and between-group differences. Data were analysed using SPSS Version 26 (IBM SPSS Statistics, USA).

## Results

A total of 25 individuals completed videoconference PR in the evaluation period and were matched to 25 individuals who completed in-person PR. Baseline characteristics are reported in Table 1. There were no significant between-group differences at baseline, although individuals in the in-person PR group had a higher number of chest infections requiring pharmacological management in the previous 12 months.

**Table 1.** Baseline characteristics.

Variable	Video PR (*n* = 25)	In-person PR (*n* = 25)	*p*-value
PSM variables			
Gender (female)	14 (56%)	15 (60%)	0.77
Age (years)	68.0 (63.0, 75.5)	68.0 (62.5, 76.5)	0.79
LINQ	8.4 (5.0)	7.7 (4.2)	0.79
Baseline variables			
BMI (kg/m^2^)	26.3 (22.1, 29.7)	27.1 (25.0, 32.0)	0.16
Smoking status			
Current	4 (16%)	5 (20%)	0.57
Former	20 (80%)	20 (80%)	
Never	1 (4%)	0 (0%)	
Smoking pack years	30.0 (20.0, 49.0)	42.5 (20.0, 62.3)	0.21
No. Hospital admissions in previous 12 months	0 (0, 1)	0 (0, 1)	0.61
No. Chest infections requiring pharmacological treatment in previous 12 months	0 (0, 1)	2 (1, 4)	<0.01
Long-term oxygen therapy	0 (0%)	3 (12%)	0.07
Ambulatory oxygen therapy	0 (0%)	3 (12%)	0.07
MRC	3.0 (1.0)	3.4 (1.1)	0.30
CRQ-total	78.8 (21.8)	70.8 (21.6)	0.14
ISW (m)	290 (170, 460)	200 (110, 330)	0.09

Baseline data reported as number (percentage), mean (standard deviation) or median (25%, 75%). Abbreviations: BMI: Body Mass Index; CRQ: Chronic Respiratory Questionnaire; ISW: Incremental Shuttle Walk Test; LINQ: Lung Information Needs Questionnaire; MRC: Medical Research Council Dyspnoea Scale; PR: Pulmonary Rehabilitation.

Following PR, both groups achieved statistically significant improvements in the LINQ (Table 2). This improvement exceeded the minimal important difference of −1 with 76% (*n* = 19) and 80% (*n* = 20) of participants achieving clinically significant improvements in videoconference and in-person PR respectively.^3^ Although the in-person PR group achieved a greater improvement in LINQ compared to videoconference PR, there was no significant within-group difference.

**Table 2.** Response to PR.

Variable	Within-group difference		Between-group difference	
	Videoconference PR	In-person PR		*p*-value
LINQ	−3.2 (−4.7 to −1.6)*	−3.0 (−4.5 to −1.4)*	0.2 (−2.0 to −2.4)	0.85
MRC	−0.3 (−0.1 to 0.7)	−0.5 (−1.0 to −0.9)*	−0.2 (−0.7 to 0.3)	0.43
CRQ-total	6.8 (1.1 to 12.5)*	20.2 (12.2 to 27.9)*	13.4 (4.2 to 22.7)	<0.01*
ISW	20 (−20, 60)	50 (0, 75)*	30 (20, 15)	0.11

Data reported as mean (95% confidence interval) change or median (25%, 75%) change. *indicates *p*-value <.05. Abbreviations: CRQ: Chronic Respiratory Questionnaire; ISW: Incremental Shuttle Walk Test; LINQ: Lung Information Needs Questionnaire; MRC: Medical Research Council Dyspnoea Scale; PR: Pulmonary Rehabilitation.

The in-person PR group achieved statistically and clinically significant improvements in MRC, CRQ and ISW. In contrast, videoconference PR resulted in statistically significant improvements in CRQ, but not MRC and ISW (Table 2). There was a statistically significant between-group difference in CRQ favouring in-person PR, but not MRC and ISW.

## Discussion

This service evaluation is the first to demonstrate that videoconference PR may be associated with similar improvements in knowledge attainment to in-person PR. Of note, 76% and 80% of participants achieved clinically significant improvement in LINQ scores in videoconference and in-person PR respectively. Nonetheless, given the small number of participants, future research should confirm these findings.

Limited research has been conducted on the effect of the structured education component of PR compared to exercise, with studies demonstrating a variable impact on knowledge attainment. However, a previous service evaluation demonstrated that structured education, delivered using pre-recorded videos as used in this evaluation, was associated with improvements in LINQ in people with COPD attending in-person PR in a community setting (mean (standard deviation) change: −4.3 (0.5)),^4^ which provides a measure of confidence in these results.

Although the results highlight the potential of PR to have a positive impact on knowledge attainment, it is not possible to ascertain what aspect of the programme leads to this improvement, and this requires further investigation.

A secondary finding of this study is that although there were no significant between-group differences, videoconference PR was not associated with improvements in breathlessness and exercise capacity, compared to in-person PR. Despite significant improvements in CRQ in the videoconference PR group following PR, there was a significant between-group difference that favoured in-person PR. These results contrast with previous data,^2^ and may be due to the small sample size and no propensity score matching for baseline MRC, ISW and CRQ data. Although there were no significant between-group differences in these variables at baseline, differences were evident for example median (25th, 75th centile) ISW: videoconference 290 m (170, 460); in-person 200 m (110, 330).

## Strengths and Limitations

The exercise and education components of PR were delivered in line with national guidelines, including delivery of the recommended structured education topics by a multidisciplinary team. However, as previously noted the small sample size may bias the results, which should therefore be corroborated by future research. The LINQ domain scores were not calculated; therefore, it was not possible to explore which knowledge domains improved in response to PR. Furthermore, the LINQ only measures knowledge attainment but no other aspect of health education such as health beliefs, behaviours and outcomes, which should be investigated in future research. Aspects of the impact of education could also be measured through mastery of skills (e.g. Chronic Respiratory Questionnaire), behavioural change i.e. self-efficacy (e.g. Self-Efficacy for Managing Chronic Disease 6-Item Scale) or patient activation (Patient Activation Measure).

In conclusion, this service evaluation indicates that videoconference PR may be associated with similar improvements in knowledge attainment as in-person PR, but this requires corroboration.
